# Phyloepidemiological Analysis Reveals that Viral Divergence Led to the Paucity of Simian Immunodeficiency Virus SIVmus/gsn/mon Infections in Wild Populations

**DOI:** 10.1128/JVI.01884-16

**Published:** 2017-02-28

**Authors:** Fabian Schmidt, Florian Liegeois, Edward J. D. Greenwood, Matthew LeBreton, James Lester, Luc Deleplancque, Martine Peeters, Avelin Aghokeng, Ubald Tamoufe, Joseph L. D. Diffo, Jean M. Takuo, Nathan D. Wolfe, Eric Leroy, François Rouet, Jonathan L. Heeney

**Affiliations:** a Lab of Viral Zoonotics, Department of Veterinary Medicine, University of Cambridge, Cambridge, United Kingdom; b UMI233/INSERM1175, IRD, and University of Montpellier, Montpellier, France; c Mosaic, Yaoundé, Cameroon; d Laboratoire de Rétrovirologie, Centre International de Recherches Médicales de Franceville, Franceville, Gabon; e Virology Laboratory CREMER-IMPM, Yaoundé, Cameroon; f Metabiota, Yaoundé, Cameroon, and San Francisco, Cameroon; g Centre International de Recherches Médicales de Franceville, Franceville, Gabon; Emory University

**Keywords:** Cameroon, Gabon, SIV prevalence, bushmeat, guenon, lentivirus, nonhuman primate, simian immunodeficiency virus

## Abstract

Human immunodeficiency virus type 1 (HIV-1) is the result of cross-species transmission of simian immunodeficiency virus from chimpanzees (SIVcpz). SIVcpz is a chimeric virus which shares common ancestors with viruses infecting red-capped mangabeys and a subset of guenon species. The epidemiology of SIV infection in hominoids is characterized by low prevalences and an uneven geographic distribution. Surveys in Cameroon indicated that two closely related members of the guenon species subset, mustached guenons and greater spot-nosed guenons, infected with SIVmus and SIVgsn, respectively, also have low rates of SIV infections in their populations. Compared to that for other monkeys, including red-capped mangabeys and closely related guenon species, such an epidemiology is unusual. By intensifying sampling of geographically distinct populations of mustached and greater spot-nosed guenons in Gabon and including large sample sets of mona guenons from Cameroon, we add strong support to the hypothesis that the paucity of SIV infections in wild populations is a general feature of this monophyletic group of viruses. Furthermore, comparative phylogenetic analysis reveals that this phenotype is a feature of this group of viruses infecting phylogenetically disparate hosts, suggesting that this epidemiological phenotype results from infection with these HIV-1-related viruses rather than from a common host factor. Thus, these HIV-1-related viruses, i.e., SIVcpz and the guenon viruses which share an ancestor with part of the SIVcpz genome, have an epidemiology distinct from that found for SIVs in other African primate species.

**IMPORTANCE** Stable virus-host relationships are established over multiple generations. The prevalence of viral infections in any given host is determined by various factors. Stable virus-host relationships of viruses that are able to cause persistent infections and exist with high incidences of infection are generally characterized by a lack of morbidity prior to host reproduction. Such is the case for cytomegalovirus (CMV) and Epstein-Barr virus (EBV) infections of humans. SIV infections of most African primate species also satisfy these criteria, with these infections found at a high prevalence and with rare cases of clinical disease. In contrast, SIVcpz, the ancestor of HIV-1, has a different epidemiology, and it has been reported that infected animals suffer from an AIDS-like disease in the wild. Here we conclusively demonstrate that viruses which are closely related to SIVcpz and infect a subset of guenon monkeys show an epidemiology resembling that of SIVcpz.

## INTRODUCTION

The lentivirus simian immunodeficiency virus from chimpanzees (Pan troglodytes) (SIVcpz) is derived from at least two monkey species (1). Phylogenetic analyses of SIVcpz revealed that the 5′ region and the *nef* gene are related to those of SIVrcm, from red-capped mangabeys (Cercocebus torquatus) (2). The remaining genome of SIVcpz (*vif* to *env*) is closely related to the SIVmus/gsn/mon lineage, from mustached (Cercopithecus cephus), greater spot-nosed (Cercopithecus nictitans), and mona (Cercopithecus mona) guenons (3, 4).

Numerous surveys of wild and bushmeat sub-Saharan African primates have provided a strong body of evidence that high SIV prevalence rates (20 to 80% of animals) are typical for most lentivirus-primate relationships. For example, high rates of SIV infection, relatively evenly distributed among the examined populations, have been described for red-capped mangabeys (5, 6), African green monkeys (5, 7, 8), mandrills (5, 9–11), colobuses (12, 13), and De Brazza's guenons (5, 10, 14). In contrast, extensive investigations of wild chimpanzees across their entire range of inhabitation have demonstrated that SIVcpz-infected subspecies show an epidemiology of geographically scattered reservoirs (where SIVcpz is absent from the majority [70%] of investigated troupes and areas) (15–19). It has been proposed that this epidemiology is directly related to the ability of SIVcpz to cause disease in chimpanzees (20).

**FIG 1:**
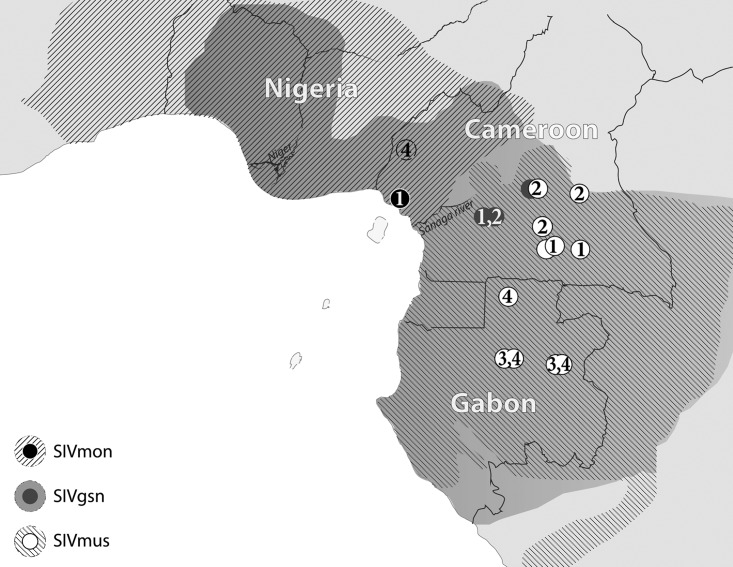
Geographic sites of documented SIVmus/gsn/mon-infected animals. Survey sites at which SIV species of the SIVmus/gsn/mon lineage were identified are indicated, corresponding to the legend on the left. Numbers in black are for SIVmon, those in gray are for SIVgsn, and those in white are for SIVmus. The hatched and shaded areas refer to geographic ranges of mustached, greater spot-nosed, and mona guenons in central Africa (www.iucnredlist.org).

In 2010, Aghokeng and colleagues reported that SIVmus in mustached guenons and SIVgsn in greater spot-nosed guenons share low prevalences of infection (up to 7% of animals carrying SIV, but an overall prevalence of around 1%), with large geographic regions in which each virus is absent (5). It therefore appears that the SIVs related to the *vpu*-to-*env* part of the SIVcpz genome share the same epidemiology as that of SIVcpz, with the caveat that extensive surveys of arboreal guenons have been carried out only in Cameroon and have not included sufficient numbers of mona guenons to estimate the prevalence of SIVmon in bushmeat (Fig. 1; Table 1). In geographically scattered surveys, we previously identified diverse SIVmus strains in Gabonese mustached guenon populations (6, 21). Here we sought to intensify regional sampling of mustached guenons and greater spot-nosed guenons in Gabon and to gain representative prevalence data for SIVmon in mona guenon populations in Cameroon. With the increased confidence that members of the SIVmus/mon/gsn lineage are generally characterized by low rates of occurrence and a scattered geographic distribution which derived from this study, we expanded our analyses to elucidate the implications that this epidemiology reveals for the evolution of this virus-host relationship.

**TABLE 1:** Prevalences identified in this and previous studies^*g*^

Country	Species or subspecies	No. of confirmed positive samples/no. of tested samples	Overall SIV prevalence (% [95% CI^*f*^])	No. on map in Fig. 1	Reference(s)
Cameroon	Cercopithecus cephus cephus	6/203^*a*^	2.9 (1.1–6.3)	1	4, 22
	C. nictitans	8/193^*a*^	4.1 (1.8–8.0)		
	C. mona	1/8^*a*^	NA^*b*^		
	C. c. cephus	9/864	1.0 (0.5–1.9)	2	5
	C. nictitans	9/859	1.0 (0.5–1.9)		
	**C. mona**	**0/244**^*c*^	**0 (0–1.5)**	**4**	
Gabon	C. c. cephus	3/50	6.0 (2.1–16.0)	3	21
	**C. c. cephus**	**3/132**^*d*^	**2.2 (0.78–6.47)**	**4**	
	**C. nictitans**	**0/92**^*e*^	**0 (0–4.0)**		

a Includes samples from pet monkeys.

b NA, not available because of an insufficient sample size (32).

c Samples were collected as dried blood spots as previously described (26, 27).

d Includes 22 freshly collected specimens and 70 specimens collected in RNAlater.

e Includes 39 freshly collected specimens and 93 specimens collected in RNAlater.

f Confidence intervals from binom.test in R.

g Data from this study are shown in bold.

## RESULTS AND DISCUSSION

**FIG 2:**
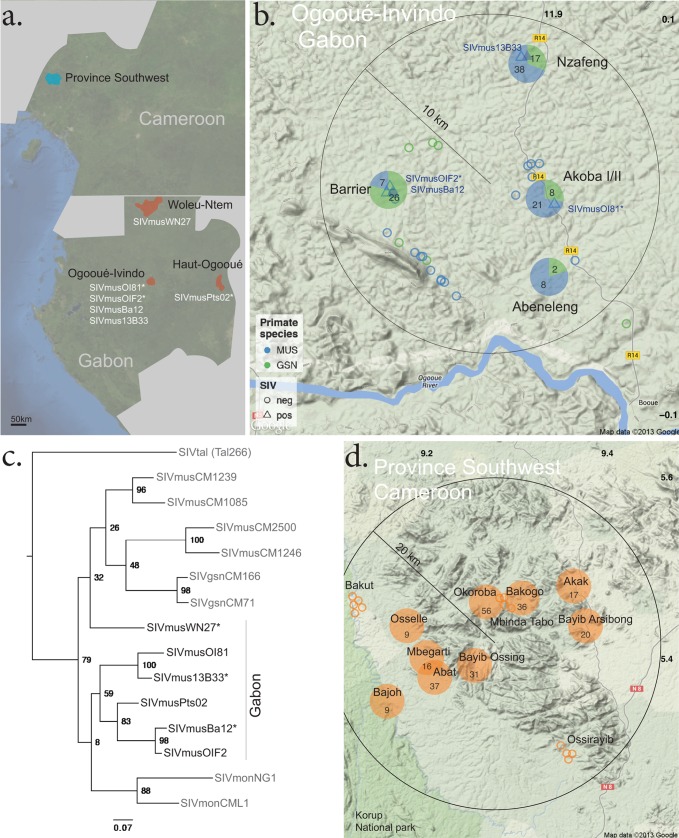
Locations of bushmeat study sites and phylogeny of SIVmus isolates. (a) Map of collection sites in central Africa. (b) Collection sites in Ogooué-Invindo, Gabon. Pie charts indicate locations at which specimens were collected by hunters. Open symbols indicate bushmeat collected fresh from the roadside. Abbreviations for species are as follows: MUS, mustached guenon; and GSN, greater spot-nosed guenon. Positive animals are indicated with triangles and negative animals with circles. (c) Phylogenetic relationships of newly identified SIVmus sequences to previously described SIVmus strains and isolates of SIVmon and SIVgsn. (d) Collection sites in Southwest Province, Cameroon. All investigated samples in the region were collected remotely by hunters and derived from mona guenons. None of these samples tested positive for SIV. Geo-plotting was carried out with R, using the ggmap package and map data from Google (29).

In 2009 and 2011, two SIVmus-infected animals (OI81 and OIF02) were identified in a central region north of the Ogooué River in the Ogooué-Ivindo province of Gabon, at sites ∼8 km apart (6, 21). Sampling during the subsequent years led to a total of 95 investigated mustached guenon individuals in a 10-km radius around this area (Fig. 2b), with two additional animals identified as carrying SIV (Ba12 and 13B33). Hence, even in this geographically highly confined area with a confirmed SIV presence, the SIV prevalence was only 4.2% (95% confidence interval, 1.36 to 10.76%), and thus within the previously reported range. Additional support for low prevalences in mustached guenons was acquired at two other collection sites. In the region of Haut-Ogooué, where isolate Pts02 was reported (21), 12 additional samples from mustached guenons tested negative. Among another 25 samples acquired in Woleu-Ntem, in the northern region of Gabon, only a single specimen tested positive (WN27). Therefore, our findings are in agreement with previous reports on the epidemiology of SIVmus (5).

Next, we used the sequences we gained as a result of our SIV confirmation to investigate relationships between these isolates. Although they are based on analysis of a short region, our findings and those of a previous report (22) suggest a complex genetic evolution of the SIVmus/mon/gsn group of viruses which was driven by interlineage recombination between SIVmus, SIVmon, and SIVgsn. For the *pol* region analyzed here, the Cameroonian strains of SIVmus and SIVgsn share a more recent ancestor to the Gabonese isolate SIVmus WN27 than to the other Gabonese isolates. Animal WN27 was identified in the north of Gabon, close to the border to Cameroon but south of the Ntem River. Interestingly, for this short region of *pol*, isolates OI81, 13B33, Pts02, Ba12, and OIF02 appear to be more closely related to sequences described for SIVmon than to those for SIVmus strains, as with our previous findings when we characterized the full-length sequences of two Gabonese SIVmus isolates (21).

Despite the interlineage recombination defining the SIV genomes in these arboreal guenon hosts, it should be highlighted that in all phylogenetic analyses, regardless of the viral gene used, SIVmus, SIVmon, and SIVgsn shared a more recent ancestor with each other than with any other monkey SIV and thus qualify to be called a monophyletic clade.

On considering the geographic locations in which these isolates were identified, we found that despite a close proximity and low prevalence, genetically diverse isolates occurred (Fig. 2c), a finding that was previously observed for this viral lineage (22). Note that despite screening of a total of 92 greater spot-nosed guenon specimens in Gabon, no infected individuals were identified.

A total of 244 specimens from mona guenons deriving from 12 sites in Southwest Province, Cameroon, were analyzed for the presence of SIV (Fig. 2d). Despite SIVmon previously being described as present in Cameroon (4) and Nigeria (3), none of these specimens tested positive.

Combined with previous studies, our data demonstrate that the epidemiology of the SIVmus/gsn/mon lineage most closely resembles that found for SIV-infected apes. It can be argued that the sampling methodologies used for surveys of great apes, which include the collection and analysis of fecal samples, and those used in this study are not directly comparable. For example, fecal sampling of ape groups includes juvenile animals, which are underrepresented in bushmeat due to size-biased hunting, leading to differences in estimations of SIV infections in bushmeat surveys compared to fecal sampling surveys. Nevertheless, the finding of an overall low prevalence, with geographically scattered reservoirs, and the occurrence of diverse strains in close geographic proximity, which represent the striking parallels between infections of great apes and the investigated subset of guenons with SIV, are unlikely to be affected by differences in sampling strategies.

**FIG 3:**
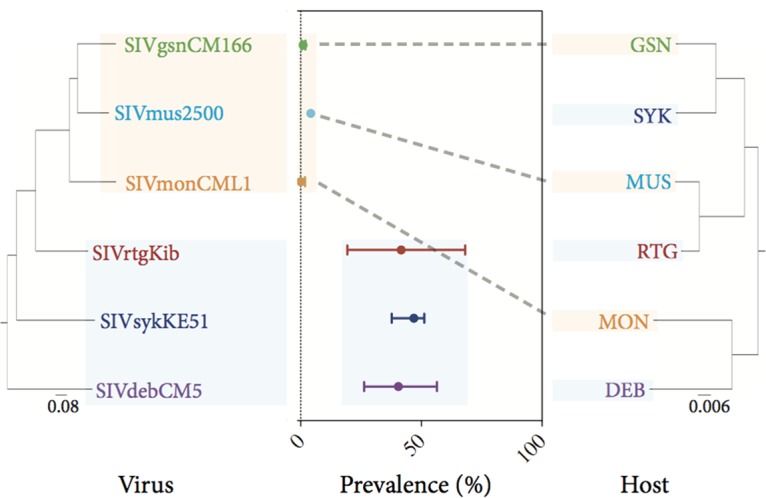
Comparative phylogeny of guenon hosts and their viruses, illustrating that parsimony in the evolution of the epidemiology observed for SIVmus/gsn/mon is given only if its characteristics evolved within the pathogen. Abbreviations for species are as follows: GSN, greater spot-nosed guenon; MUS, mustached guenon; MON, mona guenon; RTG, red-tailed guenon; SYK, Sykes guenon; and DEB, De Brazza's guenon. The mean prevalences with 95% confidence intervals (center) were plotted and correspond to the SIVs on the left. Prevalence data were derived from this study or previous studies (4, 5, 30). The tree for host species was adapted from the online resource 10kTrees, version 3 (31). The viral phylogeny is based on alignments of the envelope gene (MEGA v5).

Guenons (tribe Cercopithecini) are a species-rich group of primates. For various primates closely related to mustached, greater spot-nosed, and mona guenons, both full-length genomes of SIV isolates and prevalence data derived from bushmeat surveys are available. This enabled us to perform a comparative phylogenetic analysis of guenon hosts and isolated viruses in regard to SIV prevalence (Fig. 3).

As mentioned above, the epidemiology of the SIVmus/mon/gsn lineage is distinct from that of SIVs infecting other African monkey hosts, including closely related guenon species. For example, Sykes guenons are phylogenetically more closely related to greater spot-nosed guenons and red-tailed guenons are more closely related to mustached guenons than these species are to mona guenons. The hosts of the SIVmus/mon/gsn lineage are genetically disparate within the Cercopithecus genus. Consequently, for host evolution to account for the distinct epidemiology described for the SIVmus/mon/gsn lineage, the lineage would have to have evolved or been lost on multiple separate occasions. However, as the SIVmus/mon/gsn lineage falls within a single viral phylogeny, causality of the infecting SIV lineage with respect to this epidemiology presents a parsimonious explanation.

Multiple cross-species transmission events have shaped the SIVs we find in today's primates, and for viral mutations and variations to persist, viral isolates must enable transmission advantages over previously existing viruses. To shape host evolution toward resistance mechanisms, the virus must increase host morbidity prior to host reproduction, and exposure must be frequent enough at the population level to select for resistance/tolerance mechanisms. Crucially, the virus-host interaction studied here is not new (23). Prevalence data might therefore enable insights into the virus-host relationship. While we are confident in our conclusion that the guenon species we observed have a virus-host relationship divergent from those for other closely related monkey species, we acknowledge that the biological significance of this epidemiology calls for further attention before conclusions on the history of infection in these species can be derived. Nevertheless, due to the genomic similarities to SIVcpz and the parallels in the epidemiology, it is obvious to compare these virus-host relationships, especially given the proposed connection between the paucity of infections with SIV and a pathogenic outcome of SIVcpz infection of chimpanzees (20). If the shared epidemiology of lentiviral infections of these species is indeed due to increased pathogenicity of SIVcpz/mon/mus/gsn, then the high pathogenicity of HIV-1 can be traced to a specific part of the HIV-1 genome.

## MATERIALS AND METHODS

### Animals and samples.

Prior to our surveys, the project methodology was reviewed by the Ethical Committee for Animal Research at the Department of Veterinary Medicine, University of Cambridge (permit CR53), and authorization of governmental and provincial authorities was acquired. Strategies to study bushmeat samples varied by country to accommodate differences in local hunting habits.

In Gabon, animals are usually hunted in close proximity to the villages and sold unprocessed. After visual identification of the species, fresh samples of lymphoid organs were acquired and snap-frozen in liquid nitrogen for DNA extraction. Transection of the femoral vein or heart puncture enabled the withdrawal of blood/serum into a K3EDTA Vacutainer for serological assays. The approximate origin was acquired by communication with the hunter and with the assistance of global positioning systems (GPS).

In addition to the fresh samples, self-identified hunters volunteered to collect inguinal lymph node samples from freshly hunted nonhuman primate bushmeat in an excess volume (≥10-fold) of RNAlater (Ambion, United Kingdom). Samples were collected on subsequent field trips at intervals not exceeding 3 months. The primate species identity was confirmed using analysis of mitochondrial 12S RNA gene sequences (24, 25), applying previously established DNA extraction methods (5).

To investigate the SIV prevalence in mona guenons, a species not present in Gabon, we included dried blood spots collected in Cameroon as previously described (26, 27).

### Confirmation of SIV status.

The SIV status of all specimens was evaluated with two nested primer sets amplifying highly conserved regions of the polymerase gene as previously described (3, 21).The first round was performed with primers polis4 and PolOR or NDR1 to PolOR followed by Polis2 to Uni2 or Polis4 to Uni2, respectively. PCR amplifications were performed using a Long Expand PCR kit (Roche Applied Science) according to the manufacturer's instructions. Amplified fragments were agarose gel purified and externally Sanger sequenced. Phylogenetic analyses were carried out on viral nucleotide sequence alignments by using MUSCLE in the MEGA v7 software package. Ignoring all gaps, an alignment of 319 bp was investigated, corresponding to base pairs 3727 to 4047 of SIVmusOI81 (accession number KF304707.1), a highly conserved region. The best-fitting distance model of nucleotide substitution for each alignment was inferred using the maximum likelihood (ML) method, with goodness of fit measured by the Bayesian information criterion (BIC) in TOPALI v2.5. The model with the lowest BIC score was a general time-reversible (GTR) model with discrete gamma and invariant among-site rate variation. In the ML method (implemented in PhyML), the reliability of branching orders was tested using the bootstrap approach (100 replicates).

Serum samples collected from fresh bushmeat (from 39 mustached guenons and 22 greater spot-nosed guenons) and dried blood spots (from 244 mona guenons) were also screened for the presence of SIV antibodies by use of an in-house SIVmus/mon/gsn lineage-specific enzyme-linked immunosorbent assay (ELISA), using a synthetic SIVgsn-derived V3 loop peptide (GNKTIRNLQIGAGMTFYSQVIVGGNTRKAYC) as antigen (10) and performing the ELISA as previously described (28).

### Accession number(s).

The new sequences obtained in this study have been deposited in the EMBL database under the following accession numbers: KX825912, KX825913, and KX825914.
